# Imaging joint infections using D-methyl-^11^C-methionine PET/MRI: initial experience in humans

**DOI:** 10.1007/s00259-022-05858-x

**Published:** 2022-06-23

**Authors:** Ilona Polvoy, Youngho Seo, Matthew Parker, Megan Stewart, Khadija Siddiqua, Harrison S. Manacsa, Vahid Ravanfar, Joseph Blecha, Thomas A. Hope, Henry Vanbrocklin, Robert R. Flavell, Jeffrey Barry, Erik Hansen, Javier E. Villanueva-Meyer, Joanne Engel, Oren S. Rosenberg, David M. Wilson, Michael A. Ohliger

**Affiliations:** 1 Department of Radiology and Biomedical Imaging, University of California, 185 Berry Street, San Francisco, CA 94107 USA; 2 Department of Nuclear Engineering, University of California, Berkeley, CA USA; 3 Department of Orthopedic Surgery, University of California, San Francisco, CA USA; 4 Department of Medicine, University of California, San Francisco, CA USA; 5 Departments of Medicine and Microbiology and Immunology, University of California, San Francisco, CA USA; 6 Chan Zuckerberg Biohub, San Francisco, CA USA; 7 Department of Radiology and Biomedical Imaging, University of California, 505 Parnassus Ave., San Francisco, CA 94143 USA; 8 Department of Radiology, Zuckerberg San Francisco General Hospital, San Francisco, CA USA; 9 Department of Radiology and Biomedical Imaging, University of California, 1001 Potrero Ave. 1x55D, San Francisco, CA 94110 USA

**Keywords:** D-^11^C-Met, Prosthetic joint infection, Nuclear medicine, Positron emission tomography, Magnetic resonance imaging

## Abstract

**Purpose:**

Non-invasive imaging is a key clinical tool for detection and treatment monitoring of infections. Existing clinical imaging techniques are frequently unable to distinguish infection from tumors or sterile inflammation. This challenge is well-illustrated by prosthetic joint infections that often complicate joint replacements. D-methyl-^11^C-methionine (D-^11^C-Met) is a new bacteria-specific PET radiotracer, based on an amino acid D-enantiomer, that is rapidly incorporated into the bacterial cell wall. In this manuscript, we describe the biodistribution, radiation dosimetry, and initial human experience using D-^11^C-Met in patients with suspected prosthetic joint infections.

**Methods:**

614.5 ± 100.2 MBq of D-^11^C-Met was synthesized using an automated in-loop radiosynthesis method and administered to six healthy volunteers and five patients with suspected prosthetic joint infection, who were studied by PET/MRI. Time-activity curves were used to calculate residence times for each source organ. Absorbed doses to each organ and body effective doses were calculated using OLINDA/EXM 1.1 with both ICRP 60 and ICRP 103 tissue weighting factors. SUV_max_ and SUV_peak_ were calculated for volumes of interest (VOIs) in joints with suspected infection, the unaffected contralateral joint, blood pool, and soft tissue background. A two-tissue compartment model was used for kinetic modeling.

**Results:**

D-^11^C-Met was well tolerated in all subjects. The tracer showed clearance from both urinary (rapid) and hepatobiliary (slow) pathways as well as low effective doses. Moreover, minimal background was observed in both organs with resident micro-flora and target organs, such as the spine and musculoskeletal system. Additionally, D-^11^C-Met showed increased focal uptake in areas of suspected infection, demonstrated by a significantly higher SUV_max_ and SUV_peak_ calculated from VOIs of joints with suspected infections compared to the contralateral joints, blood pool, and background (*P* < 0.01). Furthermore, higher distribution volume and binding potential were observed in suspected infections compared to the unaffected joints.

**Conclusion:**

D-^11^C-Met has a favorable radiation profile, minimal background uptake, and fast urinary extraction. Furthermore, D-^11^C-Met showed increased uptake in areas of suspected infection, making this a promising approach. Validation in larger clinical trials with a rigorous gold standard is still required.

**Supplementary Information:**

The online version contains supplementary material available at 10.1007/s00259-022-05858-x.

## Introduction

Bacterial infections are major causes of morbidity and mortality, claiming millions of lives each year with rising prevalence [1–3]. Current imaging methods, whether structural (computed tomography (CT), magnetic resonance imaging (MRI), ultrasound) or functional (e.g., single photon emitting or positron emitting agents such as ^67^Gallium-citrate and 2-deoxy- 2-^18^F-fluoroglucose (^18^F-FDG), respectively), are frequently insufficient to identify early infection and often require invasive tissue sampling in order to achieve a definitive diagnosis [4, 5].

This problem is well illustrated by prosthetic joint infection (PJI) for which infection is a serious complication, affecting about 1% of knee and hip replacements [6]. Moreover, PJI is often treated with long-term broad-spectrum antibiotics with associated biofilm formation, leading to a 5–42% incidence of culture-negative PJI [7]. Rapid, sensitive non-invasive methods for distinguishing PJI from other aseptic mechanisms of failure, such as polyethylene-related particle wear and osteolysis [8, 9], remain one of the most significant challenges in this patient population [6].

This challenge has inspired numerous attempts to develop tools that can identify bacteria-specific metabolic processes rapidly and non-invasively, especially with the help of positron emission tomography (PET) [5, 10]. A recent study demonstrated the ability of 2-deoxy-2-^18^F-fluorosorbitol (^18^F-FDS), a sugar alcohol that is not efficiently metabolized by humans, to identify Enterobacterales infections [11]. Importantly, ^18^F-FDS is limited to use in gram-negative infections; there is an unmet clinical need for an agent that is sensitive to gram-positive infections.

D-methyl-^11^C-methionine (D-^11^C-Met) has been recently developed as a bacteria-specific PET tracer, based on the preferential incorporation of exogenous D-amino acids into bacteria [12, 13]. In bacteria, D-amino acids are assembled into peptidoglycan, an elastic polymer and essential component of the bacterial cell wall in both gram-positive and gram-negative organisms. It has been shown that D-^11^C-Met accumulates in infected rodent tissues that have been inoculated with either gram-positive or gram-negative bacteria with minimal background [12, 13]. A reliable D-^11^C-Met radiosynthesis has been successfully tested both in vitro and in animal infection models [12, 13]. In this study, we present for the first time the biodistribution, dosimetry and proof-of-principle clinical experience using D-^11^C-Met as a bacterial imaging agent in human subjects.

## Methods

### Study design

All human studies were approved by the University of California, San Francisco Institutional Review Board. All subjects provided written informed consent prior to participation. In order to qualify for the study, subjects were required to be over 18 years of age and be able to read and understand written informed consent documents. Patients were included if they had suspected infection based on one of the following: (1) clinical signs or symptoms, (2) blood or tissue cultures, or (3) radiographical findings. Subjects who were pregnant or breastfeeding were excluded. Healthy volunteers were recruited for dosimetry studies in response to an advertisement while potentially infected patients were identified by a health care provider in an outpatient facility and were referred to the study team. Subjects were evaluated throughout the study visit. Self-reported adverse events were recorded and graded according to Common Terminology Criteria for Adverse Events (version 4.0).

### Automated loop synthesis of D-^11^C-Met

D-^11^C-Met was prepared as previously reported, using an automated loop synthesis with > 99% enantiomeric excess [13] and using current good manufacturing practices. Briefly, the D-homocysteine precursor was either prepared from D-methionine (Sigma-Aldrich) or purchased from AChemTeck, Inc. All other reagents and materials were commercially available. Solid-phase exchange cartridges (Waters Sep Pak C-18) were conditioned with 5 mL of ethanol and 10 mL of water before use. A TRACERLab FXc-Pro synthesis module (General Electric) was modified to allow direct collection without high-performance liquid chromatography. The identity, radiochemical purity, and enantiomeric excess of D-^11^C-Met were determined by chiral high-performance liquid chromatography with gamma and ultraviolet detectors against the cold reference standards (D-methionine and L-methionine).

Radiochemical yield averaged over 11 subjects was 28.4% ± 6.3%, decay corrected to starting mass of carbon-11 labled CO_2_. Average radiochemical purity was 94.6% ± 1.56%. Molar activity was > 0.872 Ci/mmol. Taking into account the average injection volume of the radiopharmaceutical and the limit of detection of the D-Methionine in our quality control system (1 μg/ mL), we estimate that we have injected < 3 μg of cold mass into the subjects.

### PET/MRI acquisition

All scans were conducted on a simultaneous time-of-flight 3.0 T PET/MRI (Signa PET/MRI, GE Healthcare). Subjects were asked to void prior to the scan and were positioned supine with their arms at their sides. On the table, subjects were injected with D-^11^C-Met (mean administered activity, 614.5 ± 100.2 MBq, range 467.7–727.8 MBq).

#### Dosimetry scan

For dosimetry scans, a total of 6 whole body PETs were performed sequentially from vertex to mid-thighs at approximately 3, 10.5, 21, 41, 61, and 81 min post-injection, using one, two, or three minutes per bed position (in increasing duration) for six bed positions. A Dixon-based MRI scan was performed for attenuation correction at eachtime point. In addition, at time point 6, coronal and axial T_2_-weighted Single Shot Fast Spin Echo (SSFSE) and fat-suppressed axial T_1_-weighted Spoiled Gradient Echo (SPGR) sequences were performed. The images were reconstructed VPFXS-MAC (28 subsets, 3 iterations, PSF-on) and Q.Clear (b = 500).

#### Suspected infection

For patients with suspected infection, PET imaging began with a 30-min dynamic single bed position PET focused on the area of suspected infection, followed by six sequential whole-body scans from vertex to midthighs, or ankle respectively, using 5, 10, 30 60, 180, and 300 s per bed position acquisitions. During the dynamic acquisition, the matrix size was 192 × 192 except for QC b500 reconstruction, in which it was 256 x 256. During the initial dynamic 30-min PET acquisition, multi-planar MRI images were obtained of the affected and unaffected contralateral joint. During the whole-body phase of the acquisition, MRI was obtained for attenuation correction, together with whole-body T_2_-weighted SSFSE and T_1_-weighted SPGR as described for the dosimetry exam above.

### Normal tissue radiation dose estimation

Equivalent doses in each organ and effective doses were calculated using dynamic PET/MRI data from 3 healthy male and 3 healthy female subjects. Organ segmentation and activity concentration measurements were performed using ITK-SNAP (version 3.8.0, itksnap.org). Organ segmentations were performed on the brain, lungs, heart wall and contents, liver, kidneys, and urinary bladder. The activity within the remainder of the body was calculated as the activity from the entire volume minus the activity from all individually segmented organs. The percent of injected activity (%IA) was calculated for each organ and the remainder of the body at all time points as input data for curve-fitting to derive time-integrated activity coefficients (TIACs, a.k.a. residence times) using the EXM component of OLINDA|EXM version 1.1. Equivalent doses (in mSv/MBq) in organs and effective doses (in mSv/MBq) for the human adult male and female computational models were estimated. Organ and effective dose estimations were performed using OLINDA version 1.1 using The International Commission on Radiological Protection (ICRP) Publication 60 tissue-weighting factors [14] as well as OLINDA version 2.0 using ICRP Publication 103 tissue-weighting factors [15]. Data were reported as mean ± standard deviation (SD).

### Signal quantification of affected versus non-affected joint

Image analysis was performed using OsiriX (Pixmeo, inc). In order to quantify the PET signals, spherical volumes of interest (VOIs) with 5-cm diameters were drawn on axial images over the joints with suspected infection and unaffected contralateral joints. Blood pool activity was measured using 2-cm VOIs placed over either the femoral or popliteal arteries (for examinations of the hip or knee, respectively). Finally, soft tissue background was measured using 1-cm VOIs placed over unaffected muscles. Maximum standardized uptake values (SUV_max_) and peak standardized uptake values (SUV_peak_) were computed, normalized to the patient weight and activity injected. For purposes of this study, SUV_peak_ was defined to be the mean value of the tracer’s uptake within a 1-cm sphere surrounding the pixel with the highest activity [16]. Ten time points were utilized — the initial nine were extracted from the dynamic portion of the scan (1.5, 3, 5, 7, 11, 14, 17, 20, 25 min) and the final time point was extracted from the whole-body scan, which occurred after approximately 45 min. Next, statistical analysis was performed as described below to determine the difference between the D-^11^C-Met uptake in joint with suspected infection compared to contra-lateral joint, blood pool, and background.

### Kinetic modeling

A two-tissue compartment model was used for kinetic modeling. Since arterial blood sampling was unavailable, the closest major artery (femoral artery or popliteal artery, for the hip or knee, respectively) was chosen to derive image-based arterial input functions. There was no explicit partial volume correction applied; however, in order to minimize partial volume errors, VOIs for arterial input function, infected volume, and uninfected contralateral volume were placed well within the visualized PET uptake boundaries. All VOI selections (Fig. S3) and calculation was performed using PKIN module of PMOD (PMOD Technologies) [17]. The kinetic rate constants K_1_, k_2_, k_3_, and k_4_ were computed with the blood volume fraction (vB) set at 5%. For the first compartment, K_1_ represented the influx of D-^11^C-Met from the blood to the tissue, and k_2_ represented the flux leaving the tissue. For the second compartment, k_3_ represented the association between the tracer and the tissue, while k_4_ represented the dissociation (Fig. S4). The reversibility was evaluated by the magnitude of k_4_.

### Statistical analysis for patients with suspected infections

Image analysis was performed in OsiriX lite. All data were stored in an Excel sheet. Prism 9.2 (GraphPad Software Inc.) was used for statistical analysis*.* For SUV_max_ and SUV_peak_ calculation, one-way Anova was performed with Sidak’s multiple comparisons test, and was represented on a linear scale as mean ± standard error of the mean (SEM). *P* values < 0.05 were considered statistically significant for data analysis.

## Results

### Subject characteristics and safety

Six healthy volunteers (three men and three women) and five patients with suspected PJI were enrolled in the study. The mean age (± std dev) of the healthy volunteers was 51.5 ± 20 years (range 26–71 years). The mean age of patients with suspected infection was 71.2 ± 10.8 years (range 59–82 years). The mean dose administrated for all subjects was 614.5 ± 100.2 MBq, range 467.7–727.8 MBq (Tables 1 and 2). D-^11^C-Met was well tolerated in all subjects, with no adverse events recorded.

**Table 1:** Characteristics of healthy volunteers

Subject No	Gender	Age	Weight (kg)	Height (cm)	Injected activity (MBq)
1	M	27	61.2	183	727.8
2	M	59	113.4	190	519.8
3	M	58	111.1	178	572
4	F	68	93.9	175	506.5
5	F	71	49	160	550.5
6	F	26	77.5	160	703

**Table 2:** Characteristics of patients with suspected infection

Subject No	Gender	Age	Weight (kg)	Height (cm)	Injected activity (MBq)
1	F	82	103	152	570.5
2	F	81	67.1	170	467.7
3	F	59	106.6	171	710.4
4	M	61	97.5	179	721.5
5	M	73	88.9	175	710.4

### Biodistribution

**Fig. 1:**
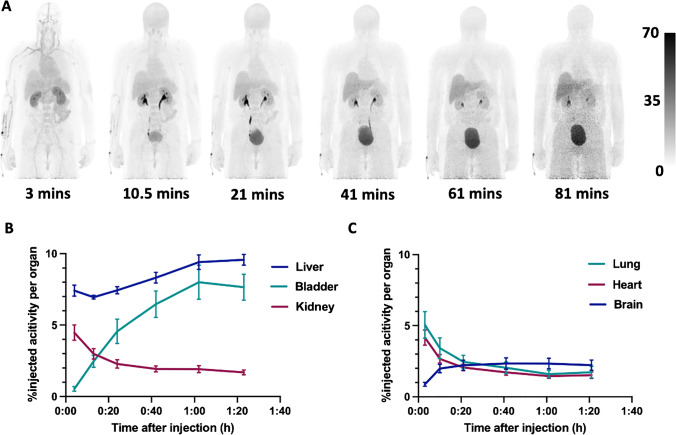
(**A**) Maximum intensity projections showing the biodistribution of D-^11^C-Met in a 59-year-old male healthy volunteer that was injected 519.8 MBq. The six successive whole-body PET scans demonstrate rapid renal clearance and a delayed liver uptake with minimal background in organs with significant human microbiomes such as the lungs and GI track as well as in target organs such as the CNS, spine, and joints. (**B**) and (**C**) show decay-corrected time-activity curves of D-^11^C-Met in the liver, kidney, and urinary bladder (**B**) and in the lung, heart, and brain (**C**) represented as mean ± SEM

After D-^11^C-Met administration, activity was initially visualized in the liver, lung, heart and the kidney during the first 4 min, rapidly declining in the lung, heart, and the kidney after an additional 20 min. D-^11^C-Met showed clearance from both urinary (rapid) and hepatobiliary (slow) pathways with a maximum value at approximately 60 min post-injection in the urinary bladder and at approximately 80 min post-injection in the liver. The remainder of the activity in the body steadily decreased over the course of the scan (Table 3). Minimal uptake was observed in the brain, with maximum uptake at approximately 40 min post injection. Decay-corrected time-activity curves of source organs are depicted in Fig. 1. Minimal background was observed in the GI tract, lungs, spine, and musculoskeletal system. Representative images from one male healthy volunteer are shown in Fig. 1.

**Table 3:** Percent injected activity (%IA) of various source organs as a function of time (mean ± SD)

Elapsed time (mins)	Brain	Lung	Heart	Liver	Kidney	Bladder	Remainder
3	0.88 ± 0.32	5.05 ± 2.29	4.16 ± 1.30	7.41 ± 0.93	4.46 ± 1.32	0.53 ± 0.39	77.49 ± 5.64
10.5	1.99 ± 0.72	3.42 ± 1.77	2.67 ± 0.72	6.95 ± 0.31	2.98 ± 0.87	2.47 ± 1.01	78.22 ± 1.54
21	2.21 ± 0.88	2.46 ± 1.11	2.06 ± 0.54	7.43 ± 0.61	2.29 ± 0.71	4.56 ± 2.08	74.68 ± 1.98
41	2.34 ± 0.96	2.04 ± 1.0	1.72 ± 0.49	8.32 ± 0.92	1.93 ± 0.51	6.46 ± 2.27	68.47 ± 8.91
61	2.33 ± 0.90	1.59 ± 0.63	1.45 ± 0.39	9.41 ± 1.24	1.92 ± 0.59	8.00 ± 2.91	66.11 ± 8.37
81	2.23 ± 0.88	1.73 ± 1.00	1.51 ± 0.57	9.57 ± 0.91	1.69 ± 0.40	7.65 ± 2.22	66.77 ± 9.89

### Equivalent radiation doses

Table 4 shows the equivalent organ doses and effective doses using ICRP 60 tissue-weighting factors. Supplementary Table S1 shows equivalent organ doses and effective doses using ICRP 103 tissue-weighting factors. Based on ICRP 60 tissue-weighting factors, the effective dose was estimated at 0.0036 ± 0.00065 mSv/MBq and 0.0046 ± 0.00066 mSv/MBq for males and females, respectively. The organs with relatively high equivalent dose were the urinary bladder, kidneys, liver, heart, and lungs. The urinary bladder showed the highest equivalent radiation dose. Table 4 also compares the measured organ doses between D-^11^C-Met and L-^11^C-Met, as reported in the literature [18]. It is worth noting that the values for both equivalent dose and absorbed dose are considered directly comparable because they are identical for ^11^C although different units for equivalent dose (mSv/MBq) and for absorbed dose (mGy/MBq) were used.

**Table 4:** Equivalent organ radiation doses and effective doses using ICRP 60 tissue-weighting factors (mean ± SD) of D-^11^C-Met in healthy volunteers, and comparison to absorbed organ doses reported for l-.^11^C-Met

Organ	Adult male equivalent dose (mSv/MBq) 73 kg	Adult female equivalent dose (mSv/MBq) 60 kg	L-^11^C-Met adult male absorbed dose (mGy/MBq)—Caucasian 1998 [18]
Adrenal glands	0.0028 ± 0.00045	0.0039 ± 0.00024	0.0037 ± 0.000043
Brain	0.0021 ± 0.00073	0.0032 ± 0.00096	0.0034 ± 0.00062
Breasts	0.0018 ± 0.00035	0.0025 ± 0.00012	0.002 ± 0.000048
Gallbladder wall	0.0030 ± 0.00047	0.0039 ± 0.00022	
LLI wall	0.0024 ± 0.00048	0.0034 ± 0.00031	0.0025 ± 0.00013
Small intestine	0.0024 ± 0.00050	0.0032 ± 0.00021	0.0045 ± 0.00053
Stomach wall	0.0024 ± 0.00046	0.0033 ± 0.00016	0.0029 ± 0.000068
ULI wall	0.0024 ± 0.00049	0.0034 ± 0.00020	0.0033 ± 0.00014
Heart wall	0.0064 ± 0.00097	0.0076 ± 0.00164	0.0076 ± 0.00029
Kidneys	0.0134 ± 0.00445	0.0153 ± 0.00321	0.011 ± 0.0011
Liver	0.0073 ± 0.00026	0.0098 ± 0.00081	0.018 ± 0.0011
Lungs	0.0057 ± 0.00237	0.0055 ± 0.00139	0.0074 ± 0.0021
Muscle	0.0020 ± 0.00042	0.0028 ± 0.00016	
Ovaries		0.0034 ± 0.00030	
Pancreas	0.0028 ± 0.00048	0.0038 ± 0.00021	0.019 ± 0.002
Red marrow	0.0019 ± 0.00037	0.0026 ± 0.00016	0.00083 ± 0.0000023
Osteogenic cells	0.0029 ± 0.00062	0.0043 ± 0.00020	
Skin	0.0016 ± 0.00035	0.0023 ± 0.00011	
Spleen	0.0024 ± 0.00045	0.0033 ± 0.00019	0.0079 ± 0.00084
Testes	0.0020 ± 0.00043		0.0022 ± 0.00014
Thymus	0.0023 ± 0.00039	0.0031 ± 0.00018	0.0024 ± 0.000057
Thyroid	0.0020 ± 0.00043	0.0026 ± 0.00010	0.0021 ± 0.00011
Urinary bladder wall	0.0155 ± 0.00159	0.0202 ± 0.01012	0.027 ± 0.0048
Uterus		0.0038 ± 0.00049	
Total body	0.0024 ± 0.00039	0.0032 ± 0.00021	
Effective dose (mSv/MBq)	0.0036 ± 0.00065	0.0046 ± 0.00066	0.0052 ± 0.00045

### Initial experience with D-^11^C-Met in patients with suspected infections

In order to gain initial experience with this new tracer, we tested it in five patients with suspected prosthetic join infection. For each subject, we provide a brief description of the clinical background, followed by qualitative observations of tracer uptake patterns. In a subsequent section, we perform quantitative analyses by comparing uptake between the affected and unaffected sides. Table 5 summarizes the clinical features of patients enrolled in the study.

**Table 5:** Clinical characteristics of patients with suspected infection

	Pain (VAS)	Tenderness	DMR*	Effusion	WBC (units/mL)	CRP (mg/L)	ESR (mm/hr)	Suspicions XR **	Previous deep culture	Current deep culture	Antibiotics prior to the scan
1	8	Y	Y	Y	5.3	2.3	17	N	*Enterococcus faecalis*	Negative	Amoxicillin
2	3	N	Y	N	7.7	19	43	Y	*Staphylococcus aureus*	Negative	TMP/SMX ***
3	9	Y	Y	Y	9	14.4	29	Y	Negative	Negative	No
4	6	Y	Y	N/A	8.8	35.7	68	Y	Positive – N/A	*Cutibacterium acnes*	TMP/SMX ***
5	N/A	N	Y	N	7.4	13.8	17	Y	*Staphylococcus aureus*	Negative	Clindamycin

^*^DMR, decreased motion range

^**^XR, X-ray

^***^TMP/SMX, trimethoprim/sulfamethoxazole

Patient 1: 82-year-old with history of bilateral knee replacements complicated by infection of the left-sided implant due to *Enterococcus faecalis,* presenting with knee pain despite prolonged antibiotic treatment. She had no fever, chills, or malaise but had decreased range of motion, mild medial and lateral joint line tenderness, and a large knee effusion. Erythrocyte sedimentation rate (ESR) and C-reactive protein (CRP) were normal, and radiographs did not show evidence of infection. However, the clinical suspicion for infection remained high, mostly due to the increasing severity of the patient’s pain as well as the complex history of her infection. The D-^11^C-Met PET/MRI scan showed mild asymmetric uptake in the tissue surrounding the suspected infection site (Fig. S1). The patient continued to rely on oral suppressive antibiotic treatment.

Patient 2: 81-year-old with history of bilateral hip replacements, complicated by the development of a draining sinus tract to her right hip, with culture positive for *Staphylococcus aureus*. She presented with right hip pain despite prolong antibiotic treatment. She had no fever, chills, or malaise. She had decreased range of motion and abductor strength, a mild elevation of CRP and ESR, and a radiograph that was suspicious for hardware loosening. D-^11^C-Met PET/MRI scan showed focal uptake around the femoral component extending along scar line laterally as well as in an area within a previously closed sinus tract (Fig. S1). The patient continued to rely on oral suppressive antibiotic treatment.

Patient 3: 59-year-old with a history of left total knee replacement that was complicated by recurrent effusions and wound drainage. She required prolonged antibiotic treatment and multiple revisions, each of which revealed no growth from intra-operative cultures. Despite the negative cultures, suspicion for infection remained high due to persistent pain, abnormal physical exam, increased CRP, and radiographs consistent with ongoing infection. The patient underwent D-^11^C-Met PET/MRI scan that showed a significant uptake in the area surrounding the painful joint (Fig. S1). Following the scan, the patient underwent an additional revision with tissue sampling. Although the cultures remained negative, swabs showed the presence of more than 10 neutrophils in 10 high-power fields, considered a specific finding for the diagnosis of PJI [19] and the patient was started on0 long term intravenous antibiotic treatment.

**Fig. 2:**
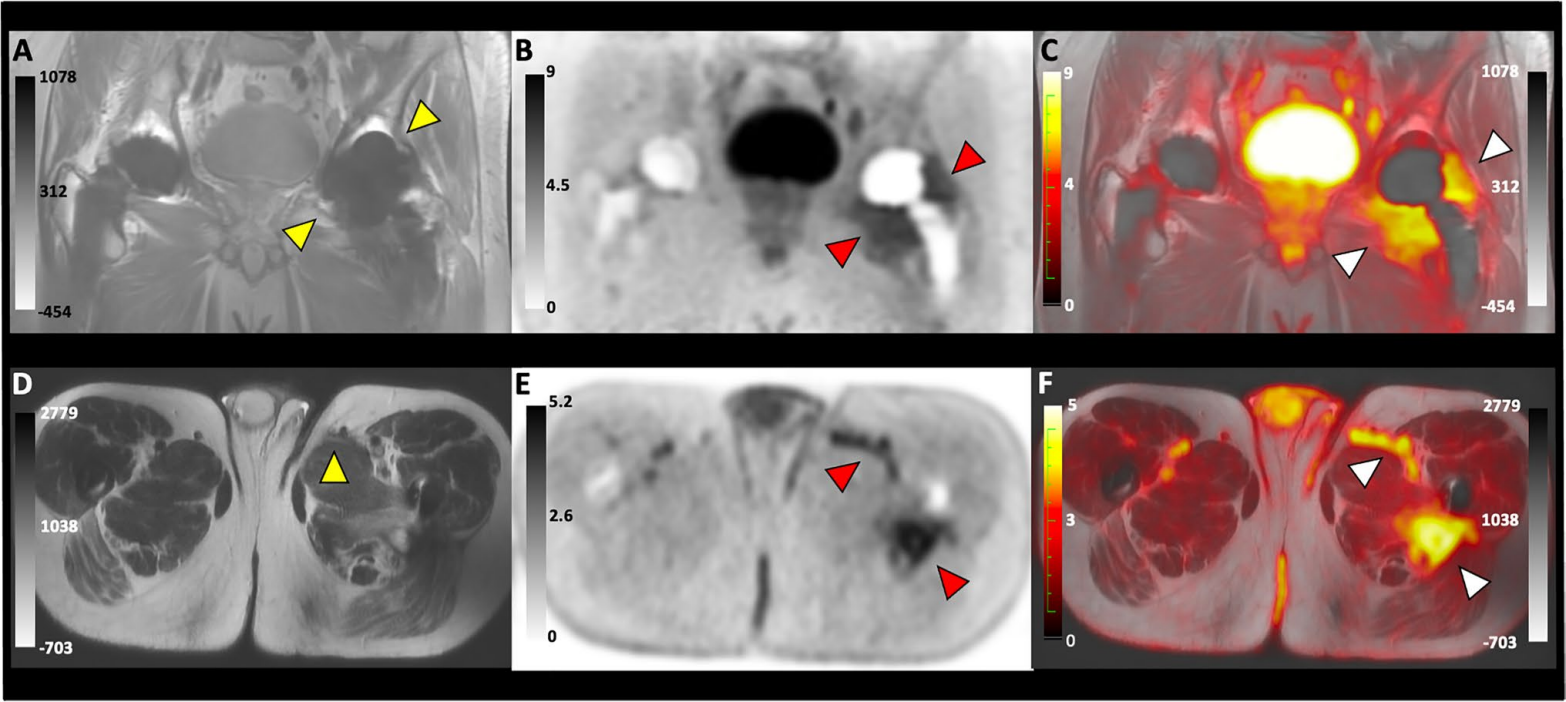
Images for D-^11^C-Met PET/MRI of a 61-year-old male with bilateral hip prosthesis and confirmed PJI of the left hip. (**A**)–(**C**) Show coronal images of MRI, PET, and Fused PET/MRI scan, respectively. The yellow arrows in (**A**) point to the infected joint. Red and white arrows in (**B**) and (**C**) point to at the area of D-^11^C-Met uptake surrounding the joint in both the PET and fused images, respectively. (**D**)–(**F**) show axial images of MRI, PET, and Fused PET/MRI scan, respectively. The yellow arrow in (**D**) points at the sinus tract in the abductor muscles. Red and white arrows in (**E**) and (**F**) point at the area of D-^11^C-Met uptake in the sinus tract in both the PET and fused images, respectively

Patient 4: 60-year-old with a history of bilateral hip replacements that was complicated by a left medial thigh abscess accompanied by sinus tract formation, requiring recurrent resections. The patient presented with increased pain in his left groin as well as recurrence of drainage from the medial proximal left thigh sinus tract. He had no fever, chills, or malaise but showed decreased range of motion, elevated CRP and ESR, and had a radiography suspicious for hardware loosening. The D-^11^C-Met PET/MRI scan showed significant uptake in the tissue surrounding the joint and in the sinus tract **(**Fig. 2, Video S1, S2**)**. Following the scan, the patient underwent a revision of the left total hip arthroplasty. Gross purulence and infected appearing material were observed throughout the proximal femur, hip joint, medial thigh musculature as well as a draining sinus tract exiting the medial proximal thigh. Culture from the left hip grew *Cutibacterium acnes*, a gram-positive rod known to cause PJI [20]. Histologic examination revealed chronic sinus tract in the left thigh and showed scarring, mixed inflammation, and granulation tissue. The patient was started on long-term intravenous antibiotic treatment.

**Fig. 3:**
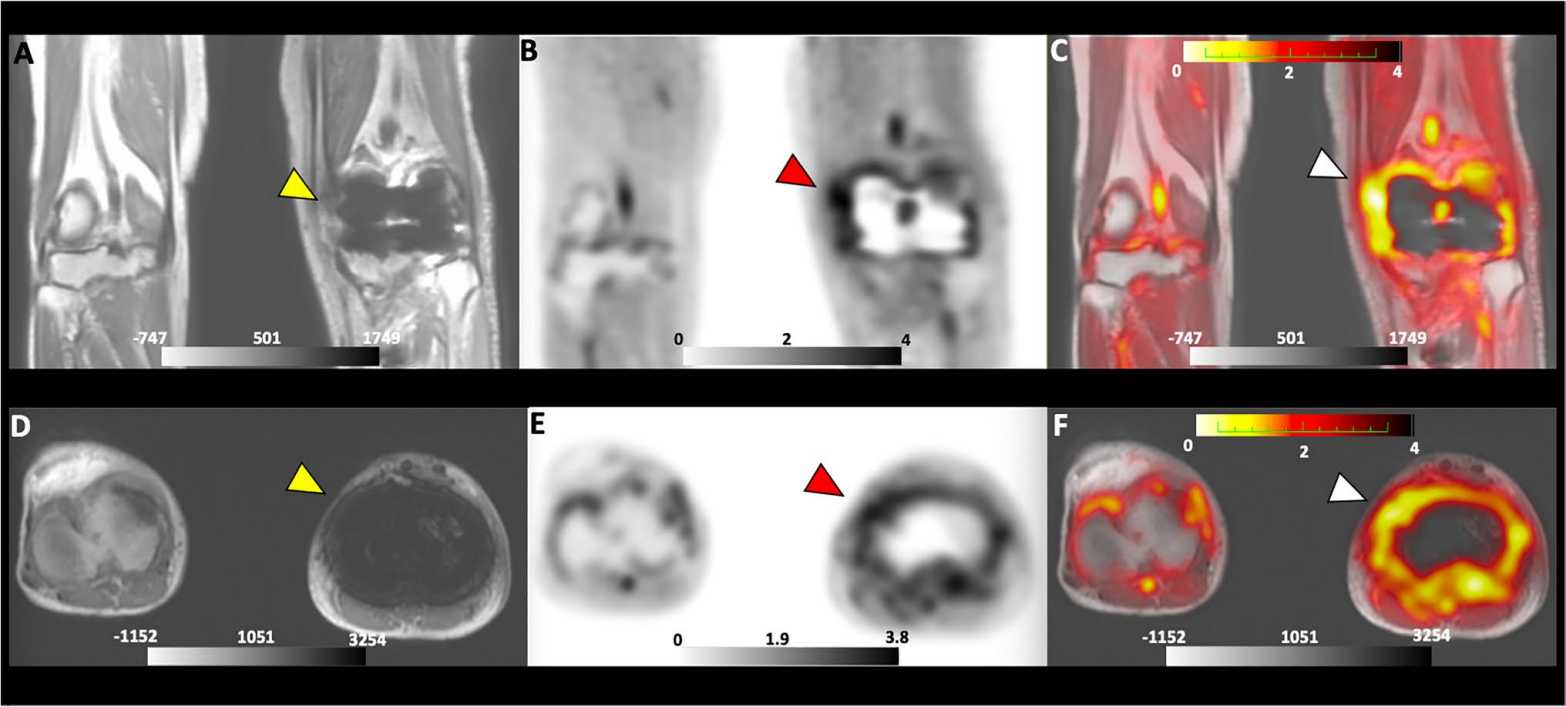
Images for D-^11^C-Met PET/MRI of a 73-year-old male with suspected PJI of the left knee. (**A**)–(**C**) show coronal images of MRI, PET, and Fused PET/MRI scan respectively. The yellow arrow in (**A**) points to the joint with the suspected PJI. Red and white arrows in (**B**) and (**C**) point to the area of D-^11^C-Met uptake surrounding the joint in both the PET and fused images, respectively. (**D**)–(**F**) show axial images of MRI, PET, and Fused PET/MRI scan, respectively. The yellow arrow in (**D**) points to the joint with the suspected infection. Red and white arrows in (**E**) and (**F**) point to the area of D-^11^C-Met uptake surrounding the joint in both the PET and fused images, respectively

Patient 5: 73-year-old with a history of multiple prior *staphylococcal* joint infections with a recent medial knee abscess positive for *Staphylococcus aureus* treated with debridement and antibiotics. He presented with ongoing left knee pain and stiffness, elevated CRP, and radiograph suspicious for hardware loosening. The patient underwent a D-^11^C-Met PET/MRI that showed significant uptake in the tissue surrounding the left knee joint (Fig. 3). Following the scan, the patient underwent a revision that was notable for arthrofibrosis associated with dense fibrotic scar tissue, and chronic inflammatory looking synovium, without signs of hardware loosening. Though the intra-operative cultures were negative, there was a high index of suspicion for infection based on his history, clinical presentation, and intraoperative report; therefore, he was placed on long-term intravenous antibiotic therapy.

### Quantitative image analysis

**Fig. 4:**
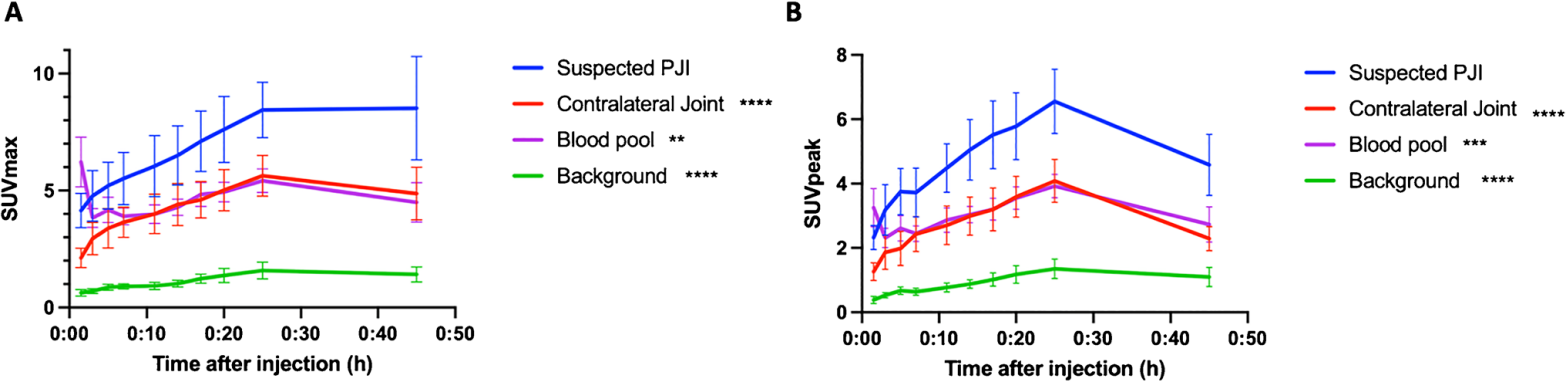
(**A**) SUV_max_ and (**B**) SUV_peak_ time course shows significant difference in D-^11^C-Met uptake in joint with suspected infection compared to contra-lateral joint, blood pool, and background. Data represented as mean ± SEM. ** = *p* < 0.01, *** = *p* < 0.001, **** = *p* < 0.0001

D-^11^C-Met showed asymmetrical uptake in areas of suspected infection compared to contra-lateral sides in all of the scanned patients. SUV_max_ and SUV_peak_ showed a significant increase in uptake in sites of suspected infection, compared to contralateral joints and blood pool, that was about 1.5 times higher throughout the scan, as well as over five times higher than the background (Fig. 4). The maximum uptake of D-^11^C-Met in the tissue with suspected infection, contralateral joint, and background tissue occurred at 25 min post-injection. The peak signal from blood pool occurred at the first dynamic time point (1.5 min). The peak SUV_max_ was approximately 1.5 times higher on the affected side compared to the unaffected side (8.5 ± 2.6 vs. 5.6 ± 1.9, *p* < 0.0001). SUV_max_ in the affected joint was more than five times greater than blood pool.

Kinetic modeling was performed for patients with bilateral hip prostheses PJI (patients 2 and 4). A two-tissue compartment model was used (Table 6, Figs. S3, S4) in which the femoral arteries were used as an image-based arterial input function and the tissue was assessed for bound and not bound radiotracer. Four kinetic rate constants K_1_*,* k_2_*,* k_3_*,* and k_4_ were determined. k_4_ was found to be greater than zero (Table 6), suggesting that the tracer was reversible and not permanently bound in the tissue [21]. However, the region with the suspected infection was found to have a larger distribution volume (K_1_/k_2_*(1 + k_3_/k_4_)) compared to the non-affected prosthetic joint on the contralateral side and it also demonstrated higher binding potential (k_3_/k_4_) for the joint with the suspected infection compared to the non-affected prosthesis [22].

**Table 6:** Kinetic modeling for D-^11^C-Met in patients with suspected hip PJI

	*K*_1_	*k*_2_	*k*_3_	*k*_4_	Distribution volume	Binding potential
Patient 2 (suspected infection)	0.33	0.358	0.225	0.457	1.379	0.493
Patient 2 (contra-lateral joint)	0.034	0.076	< 0.001	5.122	0.446	< 0.001
Patient 4 (suspected infection)	0.488	1.082	1.234	0.569	1.43	2.169
Patient 4 (contra-lateral joint)	0.019	0.053	0.004	6.431	0.374	< 0.001

## Discussion

The development of PET tracers targeting bacteria-specific metabolism has emerged from the pressing need to provide a fast and accurate diagnosis of infection [5, 23]. In recent years, functional imaging approaches, namely PET coupled with structural techniques such as CT or MRI, have greatly enhanced the ability to detect pathologies due to higher resolution and the identification of specific metabolic processes [24]. In this study, D-^11^C-Met was synthesized via an automated process [13] allowing for a fast and reproducible method, suggesting it will be easily applied in future clinical settings. Following an injection of D-^11^C-Met, PET/MRI scan in both healthy volunteers as well as patients with suspected PJI was performed.

In dosimetry studies, the tracer showed rapid uptake by the vascular compartment, resulting in high signal in the liver, lung, heart, and the kidney immediately after injection, followed by rapid clearance from circulation and fast urinary excretion. Continued tracer accumulation was observed in the liver, possibly due to the ability of the liver to metabolize D-amino acids, by either oxidization to α- keto-methionine [25] or by direct participation in multiple physiological processes such as protein synthesis and folate metabolism [26–28]. Despite potential concern for high background uptake in organs with rich microflora, we did not observe significant background uptake in the lung or gastrointestinal tract. This likely reflects poor transit of the agent into the intestinal lumen on the time scales of our studies.

The pattern of uptake for our agent was similar to that published for its enantiomer, L-^11^C-Met, that was measured in adult males [18]. One important difference in comparing the two tracers is that D-^11^C-methionine appeared to show less uptake than L-^11^C-methionine in the pancreas, spleen, and in target components of the musculoskeletal system (such as joints).

The effective dose of D-^11^C-Met was low, estimated at 0.0036 ± 0.0006 mSv/MBq and 0.0046 ± 0.0006 mSv/MBq for males and females respectively. This dose was an order of magnitude lower compared to the effective dose of fluorinated tracers such as ^18^F-FDG and ^18^F-FDS (approximately 0.02 mSv/MBq) [29, 30] and might be explained by the short half-life of the tracer. Moreover, the previously published estimated dose of L-^11^C-Met in an adult males was 0.0052 ± 0.0004 mSv/MBq, which was almost twice that of D-^11^C-Met [18]. The highest equivalent dose from D-^11^C-Met was seen in the urinary bladder wall, a result that suggests that most of the tracer’s clearance is via the urinary system.

In order to obtain initial experience with our tracer, we tested it in five patients with suspected PJI. PJI, especially in the setting of chronic infection, often presents with non-specific signs and symptoms that make definitive clinical diagnosis challenging. This clinical scenario held true for the patients with suspected chronic PJI who were enrolled in our study. While systemic signs of infection such as fever were absent, most subjects exhibited ongoing pain, decreased range of motion, and joint effusion, which are the most sensitive clinical findings of PJI [31]. Moreover, most of the patient met the diagnostic criteria for infection as dictated by the Musculoskeletal Infection Society (MSIS) [32]. Although some of the patients lacked histopathological proof of infection or had repeatedly negative cultures, the clinical suspicion for infection remained high and they were treated with long-term antibiotics.

In this study, we showed that the uptake of D-^11^C-Met was approximately 1.5 times higher in prosthetic joints with suspected infection compared to the contralateral joints. Moreover, D-^11^C-Met showed higher distribution volume and binding potential in joints with suspected PJI compared to non-infected prosthetic joints on the contralateral side. Taken together, this data supports the ability of D-^11^C-Met to accumulate in the site of the suspected infection. Complicated PJI cases, such as the ones presented here, often lack proof of infection despite the high suspicion [7], resulting in persistent infection that often require repeated surgical revisions that pose a significant impairment to function or quality of life [33]. Patients as well as health care providers will greatly benefit from the ability to diagnose infection using a quick and non-invasive tool such as PET/MRI. Although quantitative kinetic analysis was able to yield important information about the tracer such as distribution volume and binding potential, simple measurements of overall uptake (SUV_max_ and SUV_peak_) are likely more practical for long-term clinical use. We expect that SUV_peak_ will be more reflective of the overall uptake than SUV_max_, which focusses only on the highest uptake voxel. However, more extensive clinical studies will be required to prove this.

Our study had several limitations. The patient population was small and included five patients with suspected chronic infection. Most of the patients had received long courses of antibiotics, and often had negative tissue cultures. We heavily relied on clinical features to determine whether the tracer uptake supported the possibility of infection or not. We lacked definite histopathologic or microbiologic verification in most patients. If infected, the patients may have been infected with different species of bacteria, and the study duration was not long enough to provide long-term follow-up. We were unable to obtain comparisons with alternative tracers such as ^18^F-FDG or ^99m^Tc-labled white blood cell scans.

Although our data cannot definitively establish the diagnostic utility D-^11^C-Met, the results are promising, and justify further studies to understand the tracer accumulation patterns and to provide proof of the efficacy of our tracer by scanning patients with higher bacterial burden and definite tissue diagnosis.

## Conclusion

In this study, we have administrated D-^11^C-Met to healthy volunteers and to patients with suspected infection. We were able to establish the dosimetry for whole-body D-^11^C-Met scans. D-^11^C-Met was well tolerated by both groups, showed very low effective dose highlighting its safety in humans, and had a minimal background uptake, and a fast urinary extraction. Furthermore, the agent showed increased focal uptake (SUV_max_ and SUV_peak_) in joints with suspected chronic infection compared to unaffected contralateral joints. Taken together, D-^11^C-Met shows promise for use in future clinical studies testing its performance in detecting infection.

## Supplementary Information

Below is the link to the electronic supplementary material.

Supplementary file1 (DOCX 1609 KB)

Supplementary file2 (MP4 442 KB)

Supplementary file3 (MP4 4118 KB)

## Data Availability

Data is available on reasonable request.

## Abbreviations

D-^11^C-Met: D-methyl- 11C-methionine
CT: Computerized tomography
MRI: Magnetic resonance imaging
PET: Positron emission tomography
^18^F-FDG: 2-Deoxy- 2- 18F-fluoroglucose
^18^F-FDS: 2-Deoxy-2- 18F-fluorosorbitol
PJI: Prosthetic joint infection
SSFSE: Single Shot Fast Spine Echo
SPGR: Spoiled Gradient Echo
TIACs: Time-integrated activity coefficients
ICRP: The International Commission on Radiological Protection
ESR: Erythrocyte sedimentation rate
CRP: C-reactive protein
VOIs: Volumes of interest
SUV_max_: Maximum standardized uptake value
SUV_peak_: Peak standardized uptake value
vB: Blood volume fraction
SD: Standard deviation
SEM: Standard error of the mean
