# Supplementary material for: Imaging joint infections using D-methyl-11C-methionine PET/MRI: initial experience in humans
Source: Eur J Nucl Med Mol Imaging. 2022 Jun 23;49(11):3761–71. doi: 10.1007/s00259-022-05858-x (PMC9399217; doi:10.1007/s00259-022-05858-x)
Supplement: Supplementary file 1 — Supplementary file1 (DOCX 1609 KB) [file 259_2022_5858_MOESM1_ESM.docx]

Imaging joint infections using D-methyl-^11^C-methionine PET/MRI

Ilona Polvoy^1^, Youngho Seo^1,6^, Matthew Parker^1^, Megan Stewart^1^, Khadija Siddiqua^1^, Harrison S. Manacsa^4^, Vahid Ravanfar^1^, Joseph Blecha^1^, Thomas A. Hope^1^, Henry Vanbrocklin^1^, Robert R. Flavell^1^, Jeffrey Barry^4^, Erik Hansen^4^, Javier E. Villanueva-Meyer^1^, Joanne Engel^2,5^, Oren S. Rosenberg^2,7^, David M. Wilson^1*^, Michael A. Ohliger^1,3*^

1. Department of Radiology and Biomedical Imaging, University of California, San Francisco, USA
2. Department of Medicine, University of California, San Francisco, USA
3. Department of Radiology, Zuckerberg San Francisco General Hospital, San Francisco, USA
4. Department of Orthopedic Surgery, University of California, San Francisco, USA
5. Departments of Medicine and Microbiology and Immunology, University of California, San Francisco, USA
6. Department of Nuclear Engineering, University of California, Berkeley, USA
7. Chan Zuckerberg Biohub, San Francisco, California, USA

**TABLE OF CONTENTS**

1. **Supplemental Table**

**TABLE S1**: Equivalent organ radiation doses and effective doses (mean ± SD) of D-^11^C-Met in healthy volunteers using ICRP103 tissue-weighting factors………………..……….2

1. **Supplemental Figures**

**FIGURE S1** Attenuation corrected PET images for all patients with suspected PJI and target to non-target ratio……………………………………………………………………..….4

**FIGURE S2** Imaging protocol of D-^11^C-Met……………………………..………………….…5

**FIGURE S3** VOIs selection for kinetic modeling ……………...…………………….……….6

1. **Supplemental videos**

**Video S1** 3D animation of maximum intensity projection images of patient with left prosthetic hip infection

**Video S2** Dynamic acquisition of D-^11^C-Met in patient with left prosthetic hip infection in coronal plane of MRI, PET and PET/MRI fuse images

**TABLE S1**: Equivalent organ radiation doses and effective doses (mean ± SD) of D-^11^C-Met in healthy volunteers using ICRP103 tissue-weighting factors

| Organ | Adult Male Equivalent dose (mSv/MBq)  73kg | Adult female Equivalent dose (mSv/MBq)  60kg |
| --- | --- | --- |
| Adrenal glands | 0.0038±0.00078 | 0.0045±0.00032 |
| Brain | 0.0021±0.00069 | 0.0030±0.00089 |
| Breast |  | 0.0025±0.00011 |
| Esophagus | 0.0027±0.00045 | 0.0034±0.00028 |
| Eyes | 0.0018±0.00038 | 0.0024±0.00015 |
| Gallbladder wall | 0.0036±0.00049 | 0.0039±0.00023 |
| Left colon | 0.0025±0.00053 | 0.0035±0.00024 |
| Small intestine | 0.0026±0.00054 | 0.0032±0.00022 |
| Stomach wall | 0.0027±0.00049 | 0.0034±0.00017 |
| Right colon | 0.0026±0.00053 | 0.0034±0.00018 |
| Rectum | 0.0027±0.00052 | 0.0040±0.00068 |
| Heart wall | 0.0065±0.00104 | 0.0082±0.00191 |
| Kidneys | 0.0113±0.00716 | 0.0143±0.00240 |
| Liver | 0.0078±0.00034 | 0.0099±0.00081 |
| Lungs | 0.0050±0.00197 | 0.0049±0.00114 |
| Pancreas | 0.0028±0.00051 | 0.0039±0.00021 |
| Prostate | 0.0029±0.00050 |  |
| Salivary glands | 0.0020±0.00044 | 0.0026±0.00014 |
| Red marrow | 0.0021±0.00041 | 0.0027±0.00016 |
| Osteogenic cells | 0.0017±0.00035 | 0.0021±0.00013 |
| Spleen | 0.0025±0.00051 | 0.0034±0.00021 |
| Testes | 0.0020±0.00047 |  |
| Ovaries |  | 0.0035±0.00037 |
| Thymus | 0.0026±0.00043 | 0.0033±0.00027 |
| Thyroid | 0.0022±0.00048 | 0.0027±0.00012 |
| Uterus |  | 0.0040±0.00070 |
| Urinary bladder wall | 0.0159±0.00173 | 0.0182±0.00896 |
| Total body | 0.0024±0.00045 | 0.0032±0.00024 |
| **Effective Dose (mSv/MBq)** | 0.0031±0.00051 | 0.0040±0.00061 |


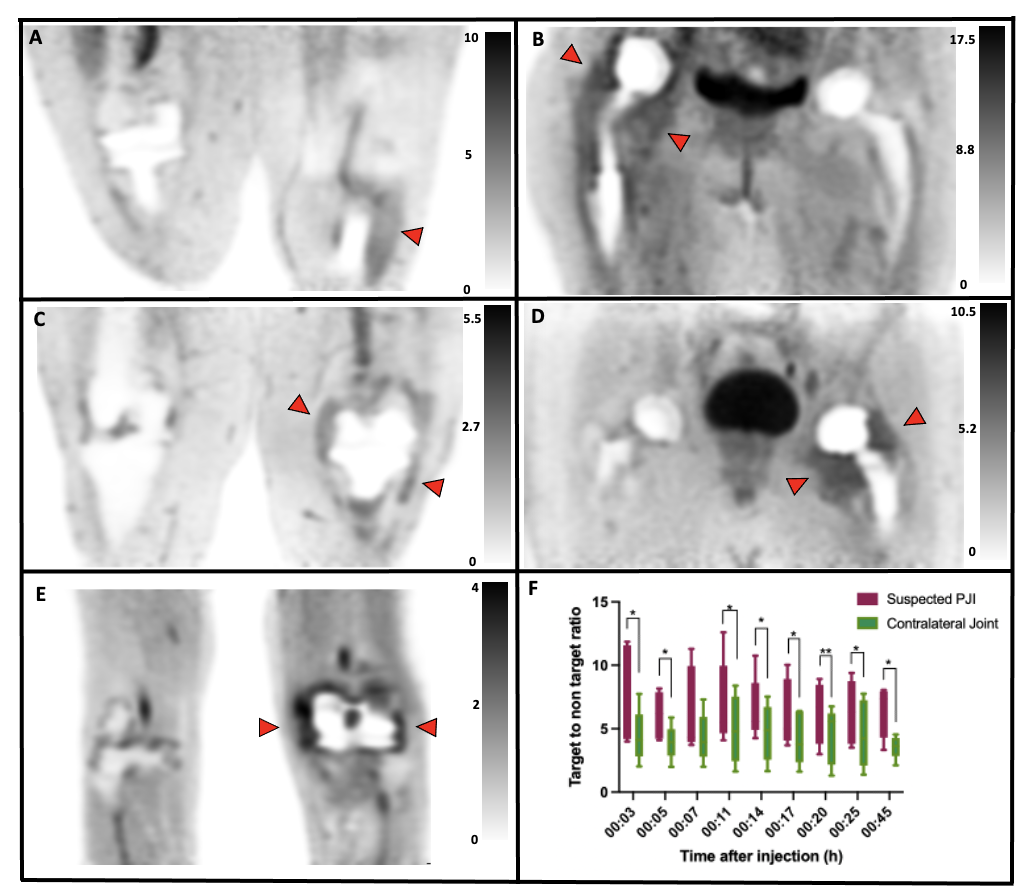


**FIGURE S1**: (A)-(E) Attenuation corrected PET images for all patients with suspected PJI: (A) an 82-year-old woman with left knee pain following total knee replacement that was later complected by hardware infection due to *Enterococcus faecalis*; (B) an 81-year-old woman with right hip pain following a total hip replacement; (C) a 59-year-old woman with left knee pain following a total knee replacement;(D) a 60-year-old male with culture positive left hip PJI; (E) a 73-year-old male with left knee pain following total knee replacement. Blue arrows in (A)-(E) show asymmetrical D-^11^C-Met uptake in sites of suspected infection. (F) Shows target to non-target ratio of site with suspected infection compared to contra-lateral joint, both normalized to muscle on the unaffected side. Paired t-test was performed to assess the statistical significance, data was represented as 5-95 percentile. * = p<0.05, ** =p<0.01.


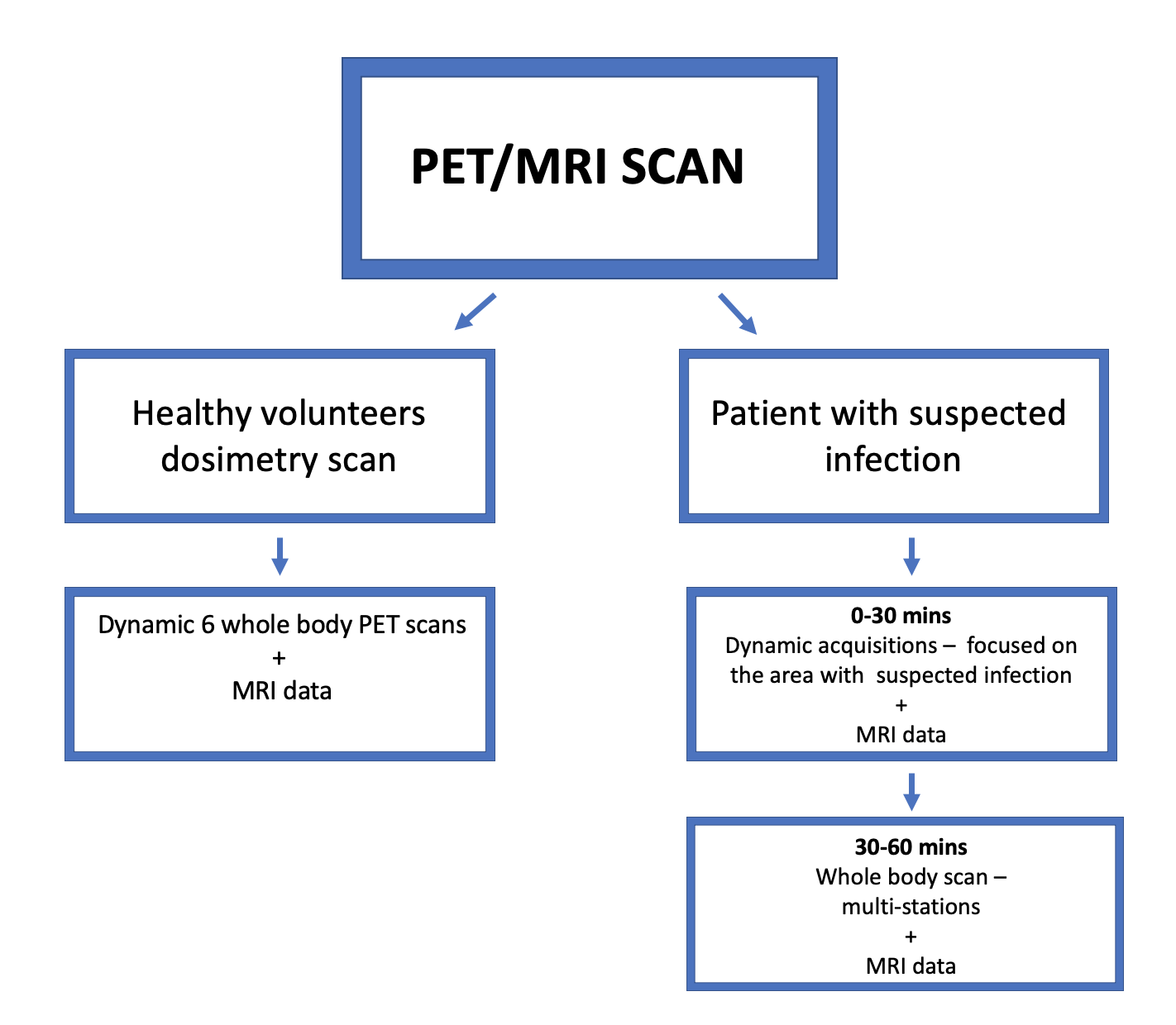


**FIGURE S2**: Imaging protocol of D-^11^C-Met in healthy volunteers and patients with suspected infection.


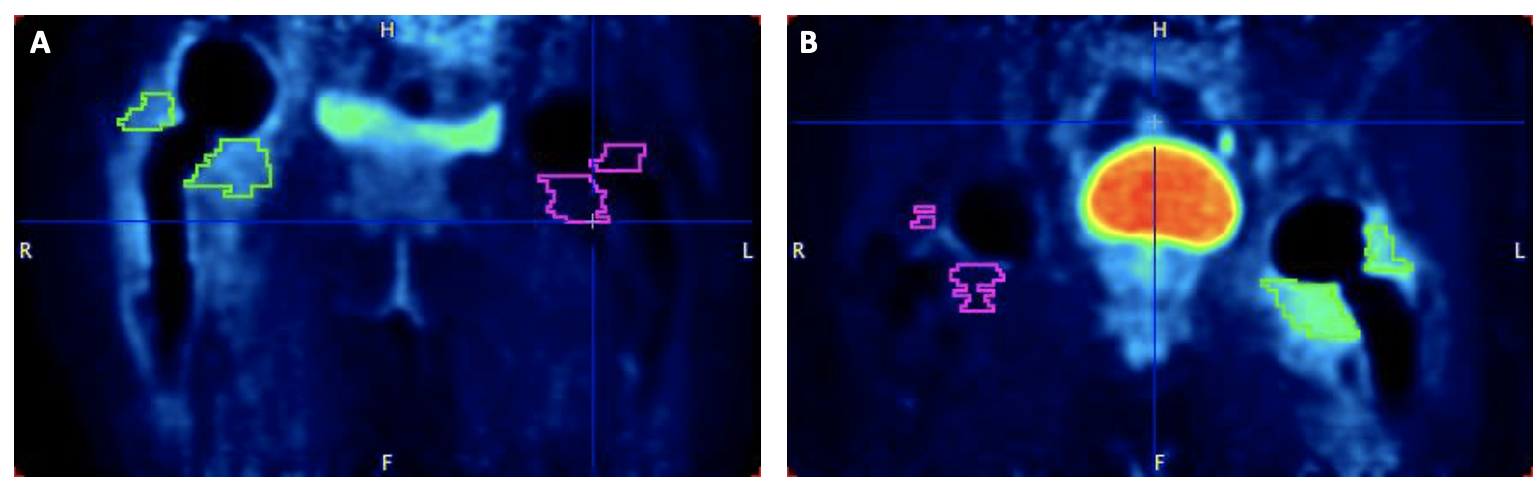


**FIGURE S3:** Selected Volumes of interests for patient number 2 with right hip suspected PJI (A) and patient number 4 with left hip PJI (B) for kinetic modeling used by PKIN module of PMOD (PMOD Technologies).


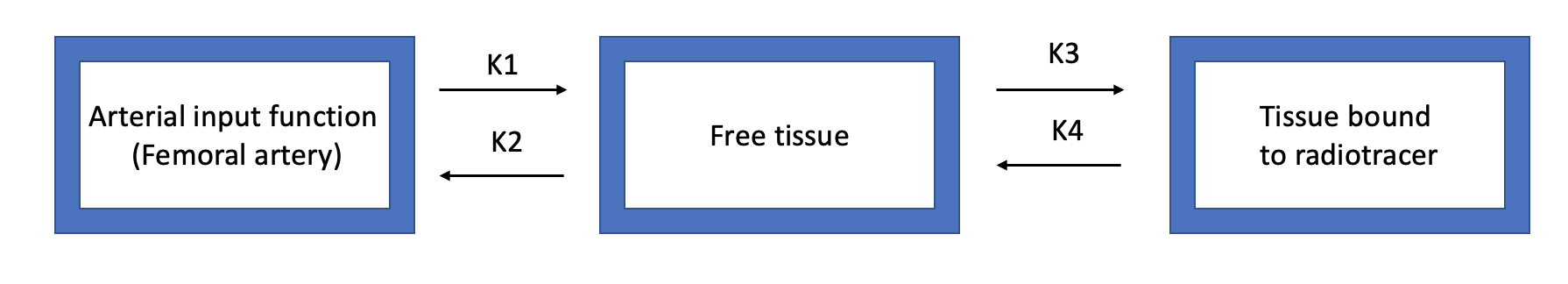


**FIGURE S4**: Illustration of the two-tissue compartment model used for kinetic modeling
